# Peripartum Cardiomyopathy: Diagnostic and Prognostic Value of Cardiac Magnetic Resonance in the Acute Stage

**DOI:** 10.3390/diagnostics12020378

**Published:** 2022-02-01

**Authors:** Alexander Isaak, Tiyasha H. Ayub, Waltraut M. Merz, Anton Faron, Christoph Endler, Alois M. Sprinkart, Claus C. Pieper, Daniel Kuetting, Darius Dabir, Ulrike Attenberger, Sebastian Zimmer, Ulrich M. Becher, Julian A. Luetkens

**Affiliations:** 1 Department of Diagnostic and Interventional Radiology, University Hospital Bonn, 53127 Bonn, Germany; alexander.isaak@ukbonn.de (A.I.); anton.faron@ukbonn.de (A.F.); christoph.endler@ukbonn.de (C.E.); sprinkart@uni-bonn.de (A.M.S.); claus_christian.pieper@ukbonn.de (C.C.P.); daniel.kuetting@ukbonn.de (D.K.); darius.dabir@ukbonn.de (D.D.); ulrike.attenberger@ukbonn.de (U.A.); 2 Quantitative Imaging Lab Bonn (QILaB), University of Bonn, 53127 Bonn, Germany; 3 Department of Obstetrics and Prenatal Medicine, University Hospital Bonn, 53127 Bonn, Germany; tiyasha_hosne.ayub@ukbonn.de (T.H.A.); waltraut.merz@ukbonn.de (W.M.M.); 4 Department of Internal Medicine II-Cardiology, University Hospital Bonn, 53127 Bonn, Germany; sebastian.zimmer@ukbonn.de (S.Z.); ulrich.becher@ukbonn.de (U.M.B.)

**Keywords:** peripartum cardiomyopathy, pregnancy, heart failure, cardiac magnetic resonance imaging, myocardial edema, mapping, strain

## Abstract

This study aimed to evaluate the diagnostic and prognostic value of cardiac magnetic resonance in acute peripartum cardiomyopathy (PPCM). A total of 17 patients with PPCM in the acute stage and 15 healthy controls were retrospectively analyzed regarding myocardial function, edema, late gadolinium enhancement (LGE), and T1 and T2 mappings (T1, T2). Echocardiographic follow-ups were performed. Functional recovery was defined as a left ventricular ejection fraction (LVEF) of ≥50%. Patients with PPCM displayed biventricular dysfunction with reduced myocardial strain parameters and left ventricular and atrial dilatation, as well as diffuse myocardial edema (T2 signal intensity ratio: 2.10 ± 0.34 vs. 1.58 ± 0.21, *p* < 0.001; T1: 1070 ± 51 ms vs. 980 ± 28 ms, *p* = 0.001; T2: 63 ± 5 ms vs. 53 ± 2 ms, *p* < 0.001). Visual myocardial edema was present in 10 patients (59%). LGE was positive in 2 patients (12%). A total of 13 patients (76%) showed full LVEF recovery. The absence of visual myocardial edema and impairment of strain parameters were associated with delayed LVEF recovery. Multivariable Cox regression analysis revealed global longitudinal strain as an independent prognostic factor for LVEF recovery. In conclusion, biventricular systolic dysfunction with diffuse myocardial edema seems to be present in acute PPCM. Myocardial edema and strain may have prognostic value for LVEF recovery.

## 1. Introduction

Peripartum cardiomyopathy (PPCM) is a rare and potentially life-threatening condition. It is defined as development of new-onset cardiomyopathy during the peripartum episode (the majority of patients present postpartum, mostly during the week after delivery) with an initial left ventricular (LV) ejection fraction (EF) of <45% and without other identifiable cause of heart failure [1,2]. The incidence of PPCM varies depending on ethnic or regional factors (e.g., ranging from 1 in 1000 to 1 in 4000 deliveries in the United States) [3,4,5]. Although different genetic, inflammatory, and immunologic hypotheses have been discussed, the exact pathogenic mechanisms of PPCM are still incompletely understood [1]. Diagnosis is primarily made by exclusion of more common differential diagnoses, such as pulmonary embolism, acute myocarditis, takotsubo syndrome, or pre-existing valvular or congenital heart disease [1]. Prognosis of PPCM is highly variable, and clinical course can vary from mild to severe [5]. The majority of patients show LVEF recovery within 6 months after diagnosis, but full LVEF recovery can be delayed, and there might even be a need for an implantable cardioverter defibrillator [1,6,7].

Although transthoracic echocardiography (TTE) is the first-line diagnostic imaging modality in case of suspected PPCM [5], cardiac magnetic resonance (CMR) is often employed in the diagnostic workup of these patients. CMR is considered the gold standard for the determination of functional and structural myocardial parameters and plays a key role in the accurate diagnosis of nonischemic cardiomyopathies, especially for the detection of acute inflammatory disease [8]. Quantitative techniques such as T1 and T2 mappings and also extracellular volume (ECV) have been shown to quantify diffuse myocardial tissue pathologies (e.g., edema) in nonischemic cardiomyopathies [9]. Furthermore, myocardial strain analysis can quantify functional alterations of the myocardium [10]. The presence of late gadolinium enhancement (LGE) in PPCM was controversially discussed in the last years; however, recent multicenter studies showed that LGE seems to be uncommon in PPCM patients (prevalence of about 4–5%) [11,12]. A few TTE-based studies in PPCM patients showed decreased strain parameters [13], which were associated with worse clinical outcome [14]. Evidence of myocardial edema was found in a few case series using T2-weighted sequences [15,16]. However, the prognostic factors and the role of myocardial edema remain poorly understood. In addition, imaging in previous studies was sometimes not performed in the acute phase of PPCM, so the full extent of myocardial alterations may have been missed.

The purpose of our study was (1) to evaluate the diagnostic value of CMR in the acute stage of PPCM and (2) to find prognostic indicators for recovery.

## 2. Materials and Methods

### 2.1. Study Population

Patients with acute PPCM and healthy control participants were included in this study. From February 2010 to January 2020, the department’s CMR registry contained 17 comprehensive scans of patients with clinical diagnosis of PPCM. All CMR scans were performed postpartum. Acute PPCM was diagnosed based on recent diagnostic criteria (occurrence of heart failure with an LVEF of <45% during the peripartum without other identifiable cause of heart failure) [1]. Serial TTE follow-ups (up to 3 years) from the clinical information system were available in 16/17 (94%) patients. An LVEF of ≥50% at TTE follow-up was defined as full LVEF recovery. CMR follow-ups were available in 6/17 patients (35%).

Due to ethical reasons, the control group consisted of healthy female controls instead of females with a normal pregnancy. All included controls were volunteers or outpatients presenting with nonspecific symptoms. All control participants had an unremarkable past medical history of cardiovascular disease. Electrocardiographic (ECG) results were unremarkable, and no cardiac risk factors were present. All control participants had normal cardiac MRI results without structural abnormalities.

### 2.2. Cardiac Magnetic Resonance Imaging

All investigations were performed on a clinical whole-body MRI system (Ingenia 1.5 Tesla, Philips Healthcare, Best, the Netherlands). A 32-channel torso coil with a digital interface was used for signal reception. Cardiac scan protocol included ECG-gated steady-state free precession cine images in short-axis, four-chamber, three-chamber, and two-chamber views. T2-weighted short-tau inversion-recovery (STIR) sequence was acquired in short-axis, two-chamber, and transversal views for the visualization of myocardial edema and for the calculation of the T2 signal intensity ratio, as previously described [8]. LGE imaging was based on a segmented inversion-recovery gradient-echo sequence and acquired in short-axis, two-chamber, and four-chamber views. Myocardial T1 and T2 maps were obtained at end diastole in apical, midventricular, and basal short-axis orientation [17,18]. Postcontrast myocardial T1 maps were performed 10 min after contrast injection. For contrast enhancement, a single bolus of 0.2 mmol/kg body weight of gadobutrol (Gadovist, Bayer HealthCare, Leverkusen, Germany) was applied. For ECV calculation, the hematocrit level on the day of the CMR scan was used. A detailed description of the CMR sequence parameters is provided in the Supplementary Materials Appendix S1 and Table S1 (online-only supplement).

### 2.3. Cardiac Image Analysis

Images were evaluated by two radiologists (J.A.L. and A.I., with 8 and 3 years of experience in CMR, respectively) using dedicated software (IntelliSpace Portal Version 10.1, Philips Medical Systems, Hamburg, Germany). Ventricular and atrial volume and mass parameters were calculated according to recent guidelines and indexed to body surface area using the Mosteller method [19]. The presence of high signal intensities on T2 STIR and on LGE images was assessed visually by consensus agreement of the two readers. The semiquantitative T2 signal intensity ratio [20] and semiquantitative enhanced volume percentage (performed in short-axis LGE images) using the full-width half-maximum technique were calculated [19]. Myocardial relaxation maps were motion-corrected using FEIR (fast elastic image registration) software (IntelliSpace Portal Version 10.1, Philips Medical Systems, Hamburg, Germany). T1 and T2 relaxation times and hematocrit-corrected ECV values (using pre- and postcontrast T1 values) were calculated as previously described [20]. Dedicated software (Image-Arena 4.6, TomTec Imaging Systems, Unterschleißheim, Germany) was used to perform feature tracking strain measurements derived from cine images in four-chamber and short-axis views to assess LV global longitudinal (GLS), circumferential (GCS), and radial strain (GRS) [10].

### 2.4. Statistical Analysis

Prism (version 8.4.3; GraphPad Software, San Diego, CA, USA) and SPSS Statistics (version 26; IBM, Armonk, NY, USA) were used for statistical analysis. The Kolmogorov–Smirnov test was applied for the assessment of normal distribution. Continuous patient characteristics are presented as mean ± standard deviation or as absolute frequency. Continuous variables between two groups were compared by using Student’s *t*-test. Due to the exploratory study design, no adjustments for multiple comparisons were made [21]. Dichotomous variables were compared by using the χ^2^ test (with a cell count > five) or Fisher exact test (with a cell count ≤ five). For intraindividual comparisons, paired Student’s *t*-test and McNemar’s test were used. Univariable and multivariable Cox regression analyses were applied to test the impact of imaging variables for the prediction of LVEF recovery. After forward selection, significant covariates with *p* < 0.05 at univariable analysis were added to a multivariable cox regression model to further fit the impact of variables. The results are presented as hazard ratios (HRs) with 95% confidence interval (95% CIs). The cohort was also binarized based on the prevalence of visual myocardial edema and based on median values of LV myocardial dysfunction (LVEF: 27%, GLS: −11.2%, GCS: −9.5%). The Kaplan–Meier method with log-rank tests was used to compare the “time to LVEF recovery” between these groups. *p* < 0.05 was defined to indicate statistical significance.

## 3. Results

### 3.1. General Characteristics

A total of 32 female subjects, 17 females with acute PPCM (33 ± 5 years) and 15 female control subjects (33 ± 8 years) were included in this study. No difference between patients and healthy controls was observed in body mass index (*p* = 0.077) or heart rate (*p* = 0.052) (Table 1). Clinical diagnosis of PPCM was made postpartum in the majority of patients (15/17, 88%; range: 1–48 days) and during the last week before delivery in only 2 patients (2/17, 12%; 1 and 3 days before delivery, respectively). In all patients, CMR was performed after delivery (range: 4–48 days; median: 10 days) and during the acute stage of disease (time between clinical diagnosis and CMR ranged from 0 to 9 days; median: 3 days). A total of 6/17 (38%) patients had cesarean section, and 1/17 (6%) patient had a twin gestation. All patients had symptoms of heart failure (NYHA class II: 5/17, 29%; class III: 9/17, 53%; class IV: 3/17, 18%) and elevated levels of serum N-terminal pro-B-type natriuretic peptide (NT-proBNP) (Table 1). Accompanying conditions were preeclampsia (4/17, 24%), HELLP (hemolysis, elevated liver enzymes, and low platelets) syndrome (2/17, 12%), uterine atony (2/17, 12%), gestational diabetes (3/17, 18%), and gestational hypertension (3/17, 18%). None had a history of cardiac disease, diabetes, or arterial hypertension before pregnancy. All patients had sinus rhythm, and 2 patients had sinus tachycardia on initial TTE. All patients were treated based on available recommendations for acute or subacute heart failure with reduced ejection fraction. Management of heart failure was individually adapted according to the clinical scenario and the course of disease. A total of 5/17 (17%) women received bromocriptine in addition to standard heart failure treatment.

### 3.2. CMR Imaging Results

**Figure 1:**
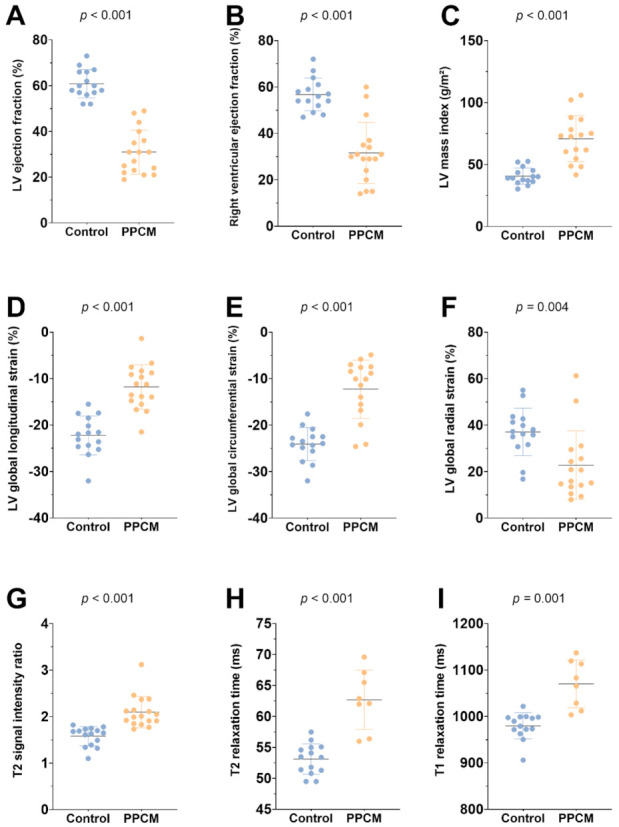
Graphs with individual plotted values show distribution of functional (**A**–**F**) and structural (**G**–**I**) cardiac MRI parameters in the control and the peripartum cardiomyopathy group (PPCM). Individual values are represented as single-colored dots. The horizontal lines show the mean values with error bars representing one standard deviation. *p*-Values were obtained using unpaired Student’s *t*-test. LV = left ventricular.

Patients with PPCM displayed reduced LVEF (31 ± 10% vs. 61 ± 6%, *p* < 0.001) and right ventricular ejection fraction (RVEF) (32 ± 13% vs. 57 ± 7%, *p* < 0.001), increased LV end-diastolic volume index (121 ± 43 mL/m² vs. 73 ± 9 mL/m², *p* < 0.001), higher left atrium volume index (75 ± 24 mL/m² vs. 40 ± 10 mL/m², *p* < 0.001), and higher LV mass index (71 ± 19 g/m² vs. 41 ± 7 g/m², *p* < 0.001) when compared with healthy controls (Figure 1). Global hypokinesia was seen in 15/17 (88%), and focal hypokinesia was seen in 2/17 (12%) patients but in none of the controls. No difference was observed in the right ventricular end-diastolic volume index (82 ± 24 mL/m² vs. 75 ± 11 mL/m², *p* = 0.300) or in the cardiac index (3.0 ± 0.7 L/min/m² vs. 3.3 ± 0.7 L/min/m², *p* = 0.228). Myocardial strain parameters were impaired in PPCM patients (GLS: −11.8 ± 4.8% vs. −22.3 ± 4.2%, *p* < 0.001; GCS: −12.3 ± 6.3% vs. −24.1 ± 3.6%, *p* < 0.001; GRS: 22.8 ± 14.7% vs. 37.1 ± 10.2%, *p* = 0.004). A total of 4/17 patients (24%) had moderate pericardial effusion (10–20 mm), and 8/17 patients (47%) had small pericardial effusion (<10 mm). Pleural effusion was present in 11/17 patients (65%). None of the patients in our cohort had evidence of LV thrombus.

Visual myocardial edema was observed in 10/17 patients (59%, controls: 0%, *p* < 0.001), and T2 signal intensity ratio was increased in the PPCM group (2.10 ± 0.34 vs. 1.58 ± 0.21, *p* < 0.001). Visual LGE was present in 2/17 patients (12%, controls: 0%, *p* = 0.484) and showed a predominantly patchy pattern of enhancement in the subepi- and midmyocardium. Quantified LGE percentages were higher in patients than in healthy controls (3.9 ± 4.7% vs. 0.6 ± 0.7%, *p* = 0.013). Myocardial native T1 relaxation times (1070 ± 51 ms vs. 980 ± 28 ms, *p* = 0.001) and T2 relaxation times (63 ± 5 ms vs. 53 ± 2 ms, *p* < 0.001) were increased in the PPCM group when compared with the control group. However, there was no significant difference in ECV between the two groups (31.7 ± 7.1% vs. 27.7 ± 3.2%, *p* = 0.235).

### 3.3. Subgroup Analyses of CMR Parameters in Patients with Follow-Up

**Figure 2:**
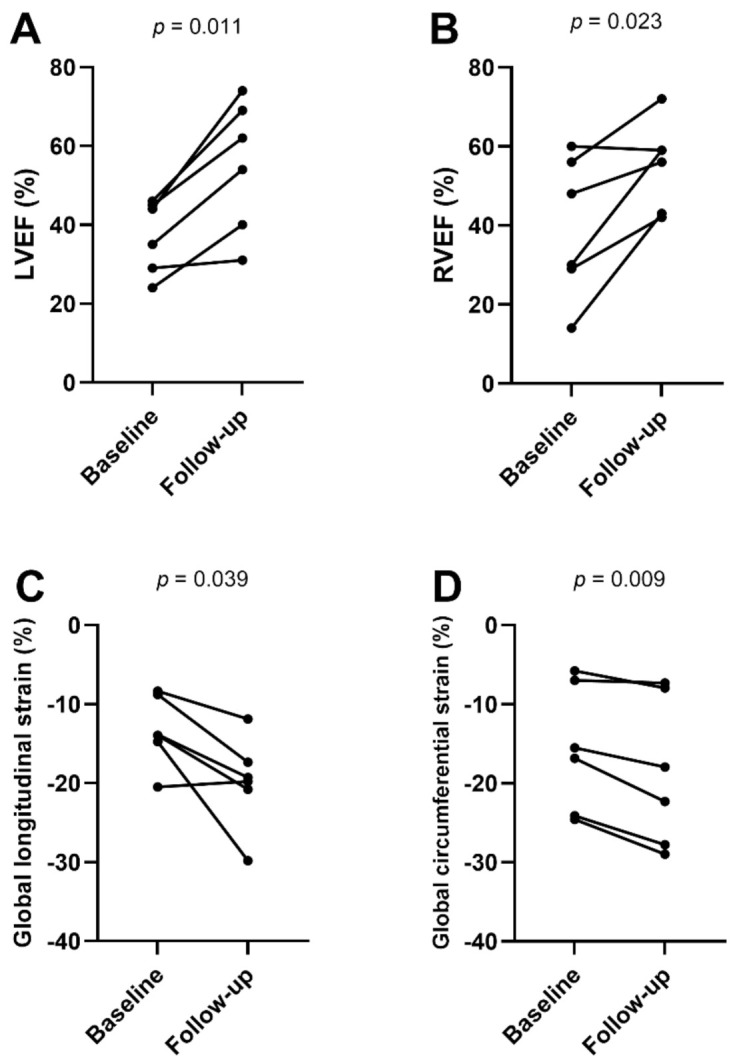
Line graphs show functional cardiac magnetic resonance parameters (**A**–**D**) at baseline (*n* = 6) and follow-up (*n* = 6). Individual values are represented by the dots at baseline and follow-up MRI. The connecting lines show the tendency of change in functional parameters over time. *p*-Values were obtained using paired Student’s *t*-test. LVEF = left ventricular ejection fraction, RVEF = right ventricular ejection fraction.

**Figure 3:**
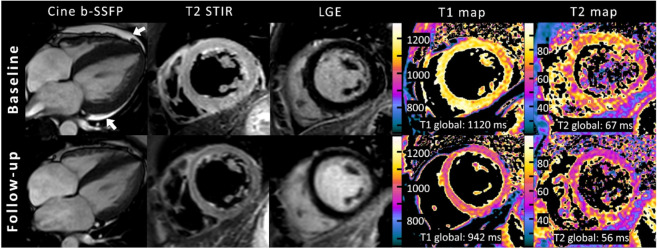
Representative example of cardiac magnetic resonance in a 32-year-old female with acute peripartum cardiomyopathy and recovery at follow-up after 2 months. Cine images (balanced steady-state free precession, b-SSFP) are oriented in horizontal long-axis view and at end systole and showed highly reduced left ventricular ejection fraction (35%) with global hypokinesia, left ventricular dilatation (left ventricular end-diastolic volume index: 118 mL/m²), and pericardial effusion (white arrows). Baseline fat-suppressed images (T2-weighted short TI inversion recovery, T2-STIR) at end diastole revealed extensive diffuse myocardial edema, which normalized at follow-up. No focal enhancement was identified on initial or follow-up late gadolinium enhancement (LGE) imaging. Quantitative mapping showed high global myocardial native T1 and T2 relaxation times at baseline MRI and normalization at follow-up.

CMR follow-up was performed in 6/17 patients (35%; median time to follow-up: 14 weeks; range: 7 to 132 weeks). Between baseline and follow-up CMR, improvement in LVEF (38 ± 9% vs. 55 ± 17%, *p* = 0.011) and RVEF (40 ± 18% vs. 55 ± 11%, *p* = 0.023) was observed (see Figure 2). There were no statistically significant differences in biventricular volumes, left atrium volumes or in LV mass index (Table 2). Interventricular septal thickness decreased on follow-up (10.5 ± 2.8 mm vs. 9.1 ± 2.0 mm, *p* = 0.047). On CMR follow-up, no visual myocardial edema was detected in any of the patients (3/6, 50% vs. 0/6, 0%, *p* = 0.25). One focal LGE lesion was still visible on follow-up CMR. Myocardial strain parameters improved between baseline and follow-up CMR (GLS: −13.5 ± 4.8% vs. −19.8 ± 5.8%, *p* = 0.039; GCS: −15.6 ± 8.1% vs. −18.7 ± 9.5%, *p* = 0.009). T1 and T2 mappings were only available in two follow-up cases and were therefore excluded from the follow-up subgroup analysis. However, the presented clinical example showed a tendency towards decreasing T1 and T2 relaxation times at follow-up, indicating myocardial recovery (Figure 3).

### 3.4. Association between Imaging Parameters and LVEF Recovery

**Figure 4:**
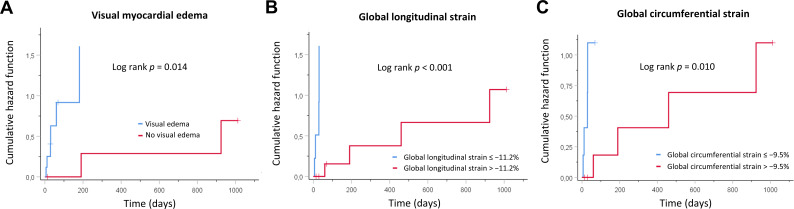
Kaplan–Meier curves showing cumulative hazard functions for left ventricular function recovery over time. Curves are given for (**A**) visual myocardial edema, (**B**) global longitudinal strain, and (**C**) global circumferential strain at initial presentation.

LVEF recovery was defined by an LVEF of ≥50% on TTE follow-up. The mean TTE follow-up time was 563 days (median: 320 days). A total of 13/17 patients (76%) recovered by the end of follow-up (mean time to recovery: 45 days; maximum time to recovery: 40 months). No patient deceased during follow-up time (all-cause mortality: 0%). Univariable Cox regression analysis showed an association between LVEF recovery and visual myocardial edema (HR = 10.17 (95% CI: 1.17, 88.65), *p* = 0.036), initial LVEF (HR = 1.13 (95% CI: 1.02, 1.25), *p* = 0.023), GLS (HR = 0.53 (95% CI: 0.34, 0.84), *p* = 0.007), GCS (HR = 0.81 (95% CI: 0.70, 0.95), *p* = 0.010), and GRS (HR = 1.10 (95% CI: 1.02, 1.18), *p* = 0.010). On multivariable Cox regression analysis, only GLS (HR = 0.51 (95% CI: 0.30, 0.85), *p* = 0.010) remained as an independent predictive variable for LVEF recovery (Table 3). According to Kaplan–Meier analysis, significantly prolonged LVEF recovery time was observed in patients without visual myocardial edema at initial presentation (840 ± 235 days vs. 145 ± 69 days, log rank *p* = 0.014), in patients with initially highly reduced GLS (663 ± 192 days vs. 51 ± 33 days, log rank *p* < 0.001), and in patients with initially highly reduced GCS (647 ± 197 days vs. 73 ± 35 days, log rank *p* = 0.010) (Figure 4), but not in patients with highly reduced LVEF at initial presentation (230 ± 181 days vs. 549 ± 166 days, log rank *p* = 0.125), in patients with bromocriptine added to standard heart failure therapy (315 ± 149 days vs. 432 ± 272 days, log rank *p* = 0.487), or in patients with concomitant preeclampsia (388 ± 270 days vs. 422 ± 178 days, log rank *p* = 0.874).

## 4. Discussion

This case-control CMR study revealed biventricular systolic dysfunction and signs of diffuse myocardial edema in the acute stage of PPCM. Prolonged myocardial T1 and T2 relaxation times in the predominant absence of LGE lesions indicate a mainly diffuse pattern of myocardial edema and emphasize the diagnostic benefit of quantitative myocardial parameters in patients with PPCM. Furthermore, the absence of myocardial edema was associated with delayed LVEF recovery. At long-term follow-up, 76% of the patients showed LVEF recovery (median time to LVEF recovery: 45 days). However, some patients showed prolonged recovery times (up to 40 months). RVEF reduction and RV dilatation were present in our PPCM cohort, indicating the presence of right ventricular involvement in the acute stage of PPCM. Furthermore, LV strain parameters, especially GLS and GCS, were markedly reduced and associated with LVEF recovery in our cohort. Impaired baseline GLS (≥−11.2%) and GCS (≥−9.5%) were associated with delayed recovery times, while no significant difference was observed for impaired baseline LVEF (≤27%). Multivariable analysis revealed GLS as an independent prognostic factor for LVEF recovery.

Recovery times in the present literature vary depending on the patient cohort and the definition of recovery. High recovery rates were described by Ersbøll et al. (85% recovered to an LVEF of ≥55% at 12 months after diagnosis) and McNamara et al. (72% recovered to an LVEF of ≥50% at 12 months), and relatively low recovery rates were observed by Mahowald et al. (37% recovered to an LVEF of ≥55% by 12 months) [12,22,23]. Various prognostic factors for long-term recovery of PPCM have been investigated in the past years. Severe LV dysfunction at initial presentation was associated with prolonged recovery times of LVEF in different studies [7,22]. However, the prognostic value of baseline LVEF alone is insufficient for differentiation between early and delayed recovery times and for indication of advanced therapies [5]. Our findings are in line with several speckle tracking echocardiography studies [13,14] and indicate a comprehensive evaluation of LV systolic dysfunction including strain parameters in addition to the assessment of LVEF only in patients with suggested PPCM. According to Haghikia et al. [24], RV dysfunction was present in our cohort of acute PPCM. Our findings support the need for a careful assessment of biventricular function and dimension at the initial presentation of patients with suspected PPCM. Since evaluation of RV parameters by echocardiography may be inaccurate, further evaluation by CMR should be considered for a comprehensive and precise assessment of right ventricular parameters in patients with suggested PPCM. The presence and role of preeclampsia in PPCM is variously described in previous studies, and there is evidence that hypertensive crisis in the presence of preeclampsia can promote LV dysfunction [12,25]. Preeclampsia was present in about a fourth of the patients in our cohort; however, no prognostic impact on LVEF recovery was found in our study.

The etiology of PPCM is still not fully understood and is suggested to be multifactorial. However, noninfectious inflammatory pathways and oxidative stress could play an important role [26]. Renz et al. described an increased T2 ratio in a small case series of acute PPCM, and Liang et al. showed increased T2 mapping values [27,28]. Other CMR studies did not detect a presence of focal or diffuse myocardial edema in PPCM patients [11]; however, imaging was partly performed beyond the acute stage of disease. In the present cohort of patients with acute PPCM, an elevation of T2 signal intensity ratio and T1 and T2 relaxation times were seen, which indicates the presence of diffuse myocardial edema. Furthermore, higher values of the interventricular septum thickness and LV mass index, which decreased on follow-up, may also be a result of myocardial edema/inflammation due to myocyte swelling and fluid accumulation [29]. However, this effect may also be contributed by physiological changes during pregnancy, which may not be completely reversed directly after birth [30]. Our findings of higher myocardial T1 and T2 relaxation times, which decreased on follow-up, are in line with the findings of Liang et al. [28] and indicate acute myocardial injury and myocardial edema, which seem to play an important role in the acute phase of PPCM [31]. Interestingly, prolonged LVEF recovery times were observed in patients without visual myocardial edema, indicating that the presence of visual myocardial edema may be a sign of a potentially reversible myocardial injury. These findings are in line with a study of inflammatory cardiomyopathies [32]. In contrast to the study of Liang et al., ECV values in our study were not significantly different from those in the healthy control group. ECV reflects the volume of cell-free heart tissue. This includes the intracapillary plasma volume (which is increased in inflammatory changes), but also the space, which is occupied by the extracellular matrix, being therefore also a surrogate for myocardial fibrosis [33]. The absence of higher ECV values in conjunction with prolonged T1 and T2 relaxation times in our study could be explained by the mainly acute stage of disease in our cohort, indicating the presence of acute myocardial injury and edema without irreversible myocardial fibrosis. LGE is a well-established marker for the assessment of myocardial fibrosis. Although some studies did report higher rates of LGE in PPCM [24], our observations of a low prevalence of focal LGE lesions in acute PPCM (prevalence of 12% with a mainly patchy LGE pattern) are in line with recent multicenter studies [11,12].

Our study has several limitations. Because of the retrospective design, clinical data assessment was limited. The single-center cohort is small due to the extremely low incidence of PPCM. Thus, the generalizability of regression models is limited, and this study should be considered to be hypothesis generating. There was no histopathological reference standard available; however, clinical use of endomyocardial biopsy has generally become very rare due to “sampling error” and periprocedural risks, and performance is especially avoided during pregnancy or the peripartum period. Due to ethical reasons, the control group consists of nonpregnant women. However, a previous study of myocardial mapping and strain analysis in healthy women during pregnancy showed LV remodeling with mild to moderate LV hypertrophy, but no evidence of functional impairment, dilatation, edema, or fibrosis of the ventricles [30].

## Figures and Tables

**Table 1:** Clinical and cardiac magnetic resonance imaging characteristics of patients with acute peripartum cardiomyopathy (PPCM) and healthy controls.

Variable	Patients with PPCM (*n* = 17)	Healthy Female Controls (*n* =15)	*p*-Value
*Clinical parameters*			
Age (years)	33 ± 5	33 ± 8	0.892
Weight (kg)	77 ± 19	67 ± 13	0.088
Height (cm)	170 ± 8	170 ± 7	0.972
Body mass index (kg/m²)	27 ± 7	23 ± 4	0.077
Heart rate (bpm)	78 ± 27	75 ± 11	0.052
NT-proBNP (pg/mL)	8792 ± 12,308	NA	-
Troponin I (ng/L)	0.12 ± 0.25	NA	-
C-reactive protein (mg/L)	15.0 ± 11.1	NA	-
White blood cells (G/L)	10.5 ± 3.7	NA	-
*CMR parameters*			
Left ventricular ejection fraction (%)	31 ± 10	61 ± 6	**<0.001**
Left ventricular end-diastolic volume index (mL/m²)	121 ± 43	73 ± 9	**<0.001**
Right ventricular ejection fraction (%)	32 ± 13	57 ± 7	**<0.001**
Right ventricular end-diastolic volume index (mL/m²)	82 ± 24	75 ± 11	0.300
Cardiac index (L/min/m²)	3.0 ± 0.7	3.3 ± 0.7	0.228
Left atrium volume index (mL/m²)	75 ± 24	40 ± 10	**<0.001**
Left ventricular mass index (g/m²)	71 ± 19	41 ± 7	**<0.001**
Interventricular septal thickness (mm)	10.3 ± 1.9	7.9 ± 1.1	**<0.001**
T2 signal intensity ratio	2.10 ± 0.34	1.58 ± 0.21	**<0.001**
Visual myocardial edema	10 (59%)	0 (0%)	**<0.001**
Visual late gadolinium enhancement	2 (12%)	0 (0%)	0.484
Late gadolinium enhancement (%)	3.9 ± 4.7	0.6 ± 0.7	**0.013**
Global longitudinal strain (%)	−11.8 ± 4.8	−22.3 ± 4.2	**<0.001**
Global circumferential strain (%)	−12.3 ± 6.3	−24.1 ± 3.6	**<0.001**
Global radial strain (%)	22.8 ± 14.7	37.1 ± 10.2	**0.004**
T1 relaxation time, native (ms)	1070 ± 51	980 ± 28	**0.001**
Extracellular volume fraction (%)	31.7 ± 7.1	27.7 ± 3.2	0.235
T2 relaxation time (ms)	63 ± 5	53 ± 2	**<0.001**

Continuous variables are given as mean ± standard deviation. Dichotomous variables are given as absolute frequency with percentages in parentheses. *p*-Values were obtained using Student’s *t*-test and Fisher exact test. NT-proBNP = N-terminal pro-B-type natriuretic peptide. Mapping parameter (T1 and T2 relaxation times and extracellular volume fraction) were available in 8 patients. Values in bold denote statistical significance.

**Table 2:** Cardiac magnetic resonance imaging characteristics of patients with acute peripartum cardiomyopathy at baseline and follow-up.

Variable	Baseline (*n* = 6)	Follow-Up (*n* = 6)	*p*-Value
Left ventricular ejection fraction (%)	38 ± 9	55 ± 17	**0.011**
Left ventricular end-diastolic volume index (mL/m²)	89 ± 28	85 ± 27	0.651
Right ventricular ejection fraction (%)	40 ± 18	55 ± 11	**0.023**
Right ventricular end-diastolic volume index (mL/m²)	66 ± 13	71 ± 15	0.370
Left atrium volume index (mL/m²)	56 ± 18	42 ± 10	0.051
Left ventricular mass index (g/m²)	61 ± 14	52 ± 8	0.176
Interventricular septal thickness (mm)	10 ± 2.8	9.1 ± 2.0	**0.047**
T2 signal intensity ratio	2.1 ± 0.3	1.7 ± 0.3	0.126
Visual myocardial edema	3 (50%)	0 (0%)	0.25
Visual late gadolinium enhancement	1 (20%)	0 (0%)	0.99
Late gadolinium enhancement (%)	4.5 ± 3.3	5.0 ± 2.6	0.363
Global longitudinal strain (%)	−13.5 ± 4.8	−19.8 ± 5.8	**0.039**
Global circumferential strain (%)	−15.6 ± 8.1	−18.7 ± 9.5	**0.009**
Global radial strain (%)	30.1 ± 21.9	30.5 ± 17.6	0.935

Continuous variables are given as mean ± standard deviation. Dichotomous variables are given as absolute frequency with percentages in parentheses. *p*-Values were obtained using paired Student’s *t*-test or McNemar’s test. Values in bold denote statistical significance.

**Table 3:** Influence of cardiac magnetic resonance imaging data for the prediction of left ventricular ejection fraction recovery in patients with acute peripartum cardiomyopathy.

Variable	Univariable Analysis		Multivariable Analysis	
	Hazard Ratio	*p*-Value	Hazard Ratio	*p*-Value
Age (per year)	0.89 (0.77–1.03)	0.116		
Body mass index (per kg/m²)	0.99 (0.90–1.09)	0.841		
LVEF (per %)	1.13 (1.02–1.25)	**0.023**		
LVEDVI (per mL/m²)	0.99 (0.96–1.01)	0.228		
LVMI (per g/m²)	1.01 (0.96–1.05)	0.790		
LAI (per mL/m²)	0.99 (0.96–1.02)	0.585		
RVEF (per %)	1.07 (1.00–1.14)	**0.036**		
RVEDVI (per mL/m²)	1.01 (0.98–1.04)	0.422		
LV GLS (per %)	0.53 (0.34–0.84)	**0.007**	0.51 (0.30–0.85)	**0.010**
LV GCS (per %)	0.81 (0.70–0.95)	**0.010**		
LV GRS (per %)	1.10 (1.02–1.18)	**0.010**		
LGE (per %)	1.05 (0.92–1.21)	0.475		
T2 signal intensity ratio	1.77 (0.25–12.30)	0.565		
Visual myocardial edema (yes/no)	10.17 (1.17–88.65)	**0.036**		

Cox regression analysis was used. Data in parentheses are 95% confidence intervals. LVEF = left ventricular ejection fraction, LVEDVI = left ventricular end-diastolic volume index, LVMI = left ventricular mass index, LAI = left atrium index, RVEF = right ventricular ejection fraction, RVEDVI = right ventricular end-diastolic volume index, GLS = global longitudinal strain, GCS = global circumferential strain, GRS = global radial strain, LGE = late gadolinium enhancement. Values in bold denote statistical significance.

## Data Availability

The data presented in this study are available on request from the corresponding author. The data are not publicly available due to privacy and ethical restrictions.

## Supplementary Materials

The following supporting information (Technical parameters, Appendix S1 and Table S1) can be downloaded at: https://www.mdpi.com/article/10.3390/diagnostics12020378/s1.
