# Supplementary material for: Peripartum Cardiomyopathy: Diagnostic and Prognostic Value of Cardiac Magnetic Resonance in the Acute Stage
Source: Diagnostics (Basel). 2022 Feb 1;12(2):378. doi: 10.3390/diagnostics12020378 (PMC8871076; doi:10.3390/diagnostics12020378)
Supplement: Supplementary file 1 [file diagnostics-12-00378-s001.zip › diagnostics-1550037-supplementary.pdf]

Supplementary Materials

Appendix S1

Technical parameters of quantitative cardiac magnetic resonance sequences:

T1 mapping and ECV

For myocardial T1 mapping, a standard, electrocardiogram synchronized 3(3)3(3)5 modified Look-Locker inversion recovery (MOLLI) acquisition scheme[17] was applied using the following parameters: time of repetition 2.2 ms, time of echo 1.02 ms, flip angle 35°, parallel imaging factor 2, acquired voxel size 1.97 x 2 × 10 mm, reconstructed voxel size 1.17 × 1.17 x 10 mm, scan duration/breath hold 15.0 s, 3 slices. Post-contrast T1 maps were performed 10 minutes after contrast injection in the same positions as pre-contrast examinations using the same imaging technique.

T2 mapping

For myocardial T2 mapping, a six-echo gradient spin echo (GraSE) sequence[16] was used with application of the following parameters: time of repetition 1 RR interval, time of echo 23.6/ΔTE = 11.8 (6Ec), flip angle 90°, parallel imaging factor 2, acquired voxel size 1.97 × 2.03 × 10 mm, reconstructed voxel size 1.03 × 1.03 x 10 mm, scan duration/breath hold 14.0 s, 3 slices.

Table S1. Exemplary sequence parameters of the cardiac magnetic resonance scan protocol.

**Parameter**	**SSFP Cine (SA)**	**Black-blood T2 STIR (SA)**	**LGE (SA)**
Field of view (mm)	350 x 350	350 x 350	360 x 311
TR (ms)<br>TE (ms)	2.8<br>1.38	2 RR intervals<br>70	3.5<br>1.71
Flip angle (°)	60	90	15
Voxel size (mm) acquired, reconstructed	1.79 x 2 x 8<br>0.99 x 0.99 x 8	1.51 x 2.43 x 8<br>0.91 x 0.91 x 8	1.65 x 1.88 x 10<br>0.9 x 0.9 x 5
Parallel imaging factor	3	2.5	2
Scan duration	1 min 24 s	1 min 36 s	27 s
Scan time/ breath-hold	13 s	8 s	12 s
Cardiac phases per RR interval<br>Shot duration (ms)	40<br>-	-<br>134	-<br>151

SSFP=steady-state free precession, STIR=short-tau inversion recovery, LGE=late gadolinium enhancement, SA=short axis view, TR=time of repetition, TE=time to echo.
